# Relative Effects of Fabry‐Perot, Mie, and Guided‐Mode Resonance in Periodic Photonic Lattice Physics

**DOI:** 10.1002/nap2.70239

**Published:** 2026-08-13

**Authors:** Yu Sung Choi, Keisuke Ozawa, Jae Woong Yoon, Junichi Inoue, Shogo Ura, Yeong Hwan Ko, Robert Magnusson

**Affiliations:** ^1^ Department of Physics Hanyang University Seoul South Korea; ^2^ Department of Electronics Kyoto Institute of Technology Kyoto Japan; ^3^ Division of Electrical, Electronics, and Control Engineering Kongju National University Cheonan South Korea; ^4^ Department of Electrical Engineering University of Texas at Arlington Arlington Texas USA

## Abstract

We investigate the physical origins of resonance effects in one‐dimensional periodic photonic lattices (PhL), focusing on the relative roles of Fabry–Perot (FP), Mie, and guided‐mode resonance (GMR) mechanisms. Using rigorous numerical simulations and modal analysis, we demonstrate that the localized field patterns commonly attributed to FP or Mie resonances arise instead from the coherent superposition of guided slab modes. By analyzing both edge‐excited and free‐space‐illuminated configurations, we show that counterpropagating guided modes form Bloch‐like standing‐wave fields consistent with observed GMR behavior. Lattices composed of Mie‐resonant particles reveal that spectral responses are governed primarily by lattice periodicity rather than intrinsic particle resonances, with perfect reflection or transmission emerging independent of the isolated‐particle resonance condition. Furthermore, designs incorporating antireflection layers and index‐matched substructures suppress FP and Mie contributions while preserving the principal resonance spectra. These results establish that resonant photonic lattice behavior is fundamentally governed by lateral leaky Bloch modes and their coupling to external radiation via GMR, providing a unified framework for understanding and designing metamaterial‐based photonic devices.

## Graphical abstract

The physical origins of resonance effects in one‐dimensional periodic photonic lattices are studied to assess the relative roles of Fabry–Perot, Mie, and guided‐mode resonance mechanisms. It is shown that lattice resonance is fundamentally governed by lateral leaky Bloch modes and their coupling to external radiation via guided‐mode resonance. This provides a unified framework for understanding photonic lattice physics.

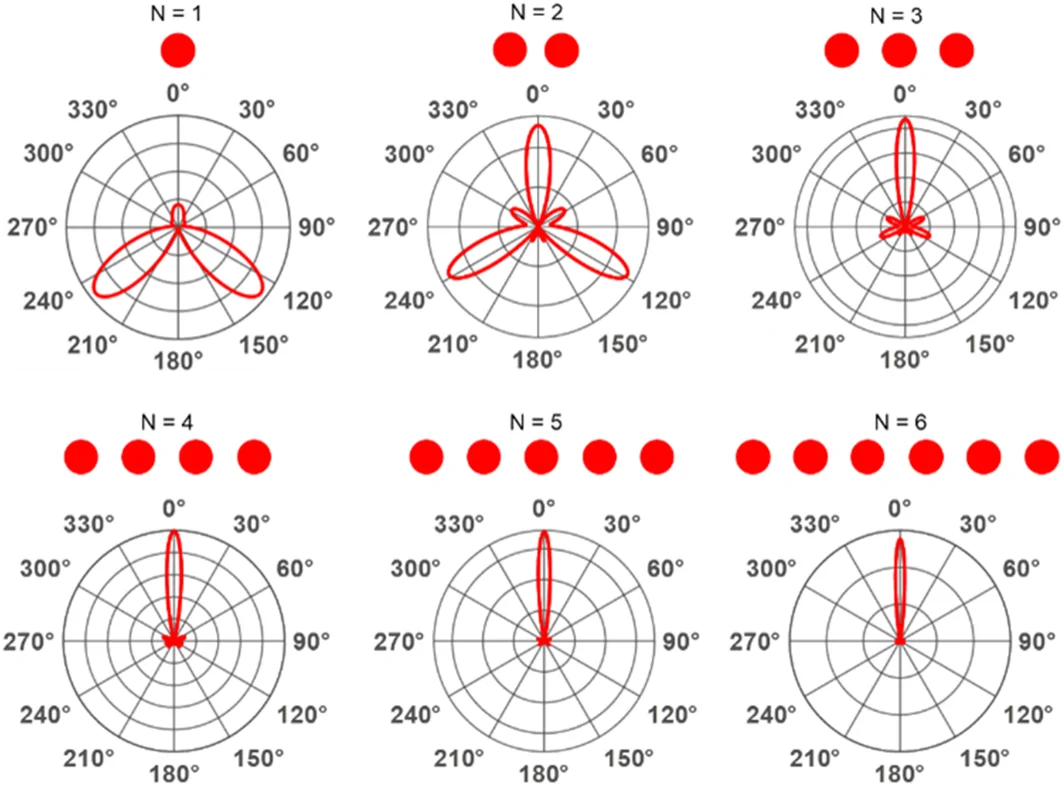

## 1 Introduction

Periodic photonic lattices support sustained resonance effects with prominent spectral features as realized with metamaterials and metasurfaces. Lateral leaky Bloch modes induced by diffractive phase matching to the lattice generate guided‐mode resonance as described in numerous articles [1, 2, 3, 4, 5, 6, 7, 8]. The descriptive term guided‐mode resonance (GMR) was coined in 1990 [9]. These resonances, which occur when external radiation couples to the leaky modes, enable diverse applications in filtering, sensing, and photonic device engineering.

In the literature, there are diverging descriptions of the basic physical operation of resonant lattices. Indeed, the properties of their spectra, including perfect reflection or transmission, are said to be governed by Fabry–Perot (FP) resonance, particle‐based Mie resonance, or guided‐mode resonance. Here, we clarify these distinct mechanisms and discuss their relevance in periodic photonic lattice physics. We show that the dominant mechanism is guided‐mode resonance also denoted as leaky‐mode resonance, Fano resonance, and nonlocal resonance.

With exact numerical methods as commercially available and broadly used in published works, the final computed solutions are typically correct as the Maxwell equations with full boundary conditions guarantee rigorous results. External spectra and local electromagnetic field distributions are thus found. Then, the physical processes behind the results are interpreted. Often, stratified field patterns observed within the lattice are interpreted as FP resonance signatures resulting from round‐trip interference between top/bottom interfaces. This relates to previous work where perfect reflection in a “high‐contrast” grating was explained as being due to strongly coupled simultaneous FP resonances of two vertically directed (± *z* propagating; see Figure 1) waveguide array modes [10, 11]. It was subsequently shown that this mechanism is not the cause of perfect reflection, it is instead being driven by excitation and reradiation of lateral Bloch modes [12]. Although vertical FP resonance does not cause the dramatic spectral signatures associated with periodic lattices, FP resonance in the effective‐medium lattice forms a background spectrum that has typically mild effects in shaping the resonance lines. Moreover, as discussed in classic textbooks, it is total internal reflection (TIR) in a film slab and subsequent FP resonance that forms the supported set of waveguide modes as per classic texts [13]. Thus, FP effects are inherent in the guided‐mode resonance picture. Accordingly, and concretely, in any resonance lattice study, there exist contributions from FP resonance in (1) forming the operative leaky modes and (2) in modulating the observed spectra via effective‐medium background fringes.

**FIGURE 1:**
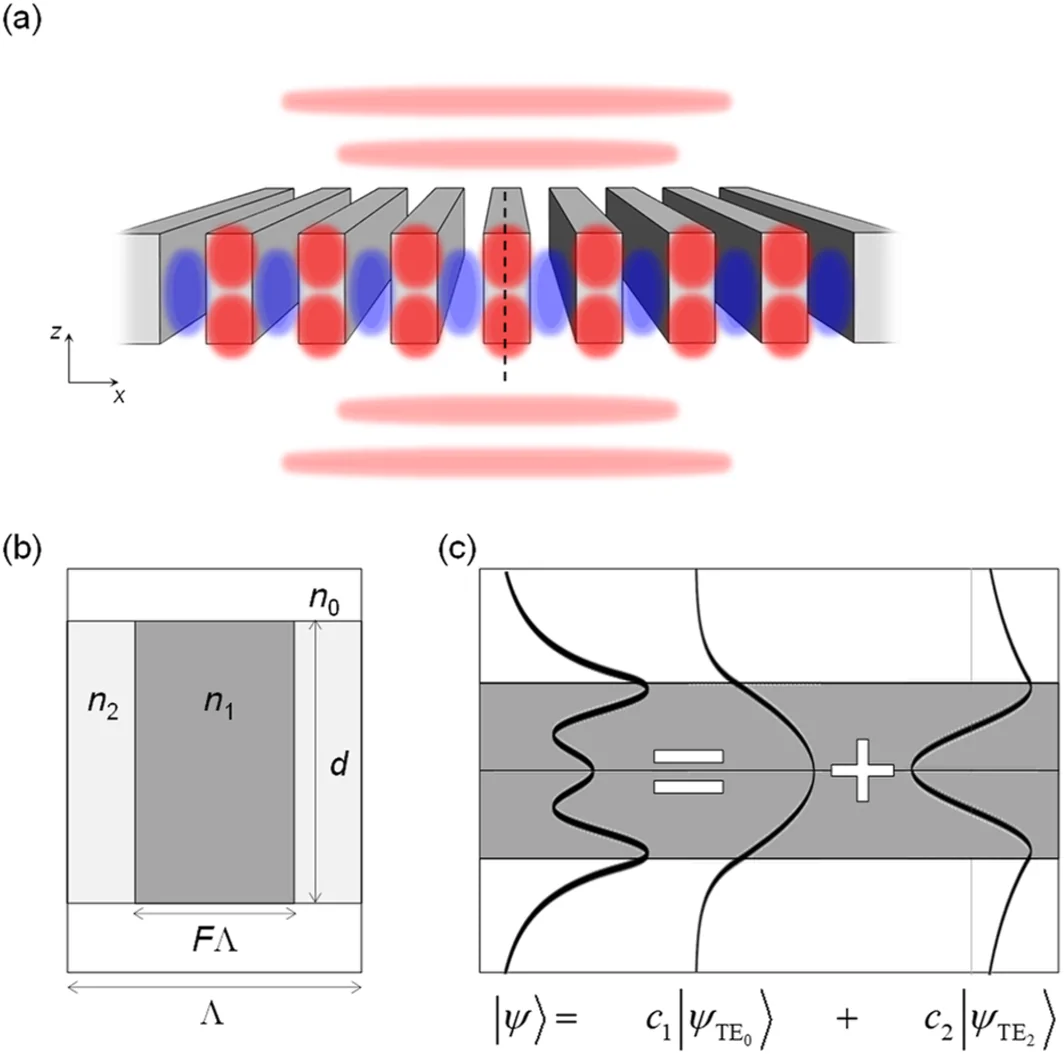
Schematic and modal features of a one‐dimensional periodic photonic lattice. (a) Schematic illustration of the GMR structure and its field distribution at resonance, showing vertically stratified fields within the high‐index regions. (b) Unit‐cell configuration of the dielectric lattice defining key parameters. (c) Vertical field profile extracted along the dashed line in (a), revealing a pattern consistent with the superposition of the even‐parity guided modes noted schematically.

However, FP interference by itself is insufficient to cause lattice resonance. Take a homogeneous film on a substrate with index *n*_*s*_ and cover medium with index *n*_*c*_. The film has index *n*_*f*_. Let *n*_*s*_ > *n*_*f*_ > *n*_*c*_. In coupling light into the film, there will be reflections at each interface and FP interference but never a guided mode. Namely, the fundamental FP mechanism is operative because partial reflections occur at both interfaces while not leading to constructive interference and sustained mode. Our contention is that the FP mechanism must result in a guided mode or else there is no resonance. Thus, FP by itself and in all cases is insufficient as this example shows. This applies also to formulations of periodic media modeled with vertical Bloch wave components and deriving isolated interface coupling coefficients [14, 15]. In applying such formulation to a periodic film, there must be the possibility of TIR at each interface in the effective medium lattice for a resonance to arise. For example, if the grating in ref. [14] is placed in a host medium with a higher index than that of the grating ridges, no resonances would exist. This is true even though all assumed processes including vertical FP effects are still in play because the formulation in models like that in [14] is independent of refractive index values. In summary, lattice resonance is guided‐mode resonance and not FP resonance.

Furthermore, as widely published, PhL perfect reflection and transmission are often attributed to Mie resonances associated with localized scattering in high‐index inclusions. Whereas FP resonance occurs via reflections between parallel planes possessing refractive‐index discontinuities, Mie resonance occurs via similar reflections but between nonparallel planes. Essentially, Mie resonance is a generalized FP resonance most often associated with isolated cylindrical and spherical particles. In a recent paper, treating a simple one‐dimensional cylindrical‐rod lattice, it is shown clearly that Mie resonance does not cause perfect reflection [16]. Rather, the spectral placement of the Bloch‐mode‐mediated zero‐order reflectance is primarily controlled by the lattice period by way of its direct effect on the homogenized effective‐medium refractive index of the lattice. Indeed, it is the effective lattice index that controls the coupling of the incident wave to the leaky Bloch mode to cause the guided‐mode resonance. Generally, perfect reflection appears away from any Mie resonance wavelength. However, when the lateral leaky‐mode field profiles approach the isolated‐particle Mie field profiles, the resonance locus tends toward the Mie resonance wavelength. The fact that the lattice fields “remember” the isolated particle fields is referred here as “Mie modal memory” [16].

In published works claiming Mie resonance for perfect reflection, the transition from the individual scattering events into a collective set of coherent diffraction orders is typically not acknowledged. In diffractive optics, say a series of slits illuminated at normal incidence, the diffraction spectrum forms numerous diffraction orders *m* that superimpose coherently into specific angular (*θ*_*m*_) directions obeying the grating equation *Λ*sin*θ*_*m*_ = *mλ*. Similarly, for a periodic array of dielectric particles, say 1D cylindrical rods, diffractive scattering occurs. The recognition of the hybridization of the individual scattering events into a diffraction spectrum whose orders can phase‐match and couple to a leaky mode and resonate is necessary to correctly claim Mie resonance as generative in guided‐mode resonance. As a final point, it is known that a lattice whose individual particles do not support Mie resonance can, in principle, generate 100% resonance reflection. This was first shown by Gomez‐Medina et al. [17]. In most of the literature claiming Mie resonance causality for perfect reflection and related spectral effects, the transition from the particle picture to the leaky‐mode picture is not explained or mentioned. This reduces the archival value of these contributions while systematically ignoring a large body of prior foundational literature.

Contextualized in this background, in what follows, we make several arguments to strengthen these views. We show how characteristics of end‐fire coupled waveguide gratings correspond to those of analogous broad‐side excited lattices. We present numerical data showing buildup of perfect reflection at a particle magnetic dipole resonance wavelength for one periodicity and conversion to perfect transmission at another periodicity using the same particle as the lattice building block. We conclude with examples of perfect reflection spectra that are essentially unaffected by the minimization of both vertical FP resonance and Mie particle resonance.

## 2 Simple‐Modal and Leaky‐Modal Field Signatures

Taking an elemental architecture, we show that the field distributions in one‐dimensional lattices can be consistently explained by the superposition of guided modes in a periodic slab. To elucidate the modal origin of the lattice fields, Figure 1 summarizes the structural configuration and corresponding field profiles. Figure 1a schematizes a lattice membrane and a representative field distribution at resonance. Vertical nodes are observed inside the high‐index regions resembling Fabry–Perot or Mie resonance signatures. Figure 1b displays the unit‐cell geometry and Figure 1c presents the field profile extracted along the dashed line in Figure 1a. The profile exhibits nodal features characteristic of the coherent superposition of the even‐parity TE_0_ and TE_2_ slab modes.

For the model in Figure 1, under end‐fire excitation, only symmetric modes with the same parity as TE_0_ can effectively couple and propagate while odd‐parity modes such as TE_1_ are suppressed. From the slab‐waveguide perspective, the grating consists of alternating high‐ and low‐index segments surrounded by a lower‐index cladding. The low‐index regions support only the TE_0_ mode, whereas the high‐index regions accommodate multiple even‐parity modes (TE_0_, TE_2_) to investigate modal interference effects. Consequently, the transverse field profile can be attributed to the superposition of even guided modes confined within the high‐index segments. This explanation is consistent with the observed nodal features and is further supported by modal decomposition analysis.

For quantitative analysis, we model a dielectric lattice with refractive indices *n*_1_ = 2, *n*_2_ = 1.44 and *n*_0_ = 1, a period *Λ* = 940 nm, thickness *d* = 1 μm, and fill factor *F* = 0.5. With these parameters, the low‐index region sustains only TE_0_, whereas both TE_0_ and TE_2_ exist in the high‐index region naturally producing modulated fields through modal overlap.

The photonic band structure in Figure 2a shows the second stop band near the *Γ* point (*k* = 0), calculated with the Kazarinov‐Henry coupled‐mode model [18, 19]. The upper band supports leaky modes with in‐plane symmetric field profiles. We investigate the resonant behavior of the leaky mode at *k* = 0 (red dot). The Bloch eigenstate calculated by the finite‐element method (FEM) exhibits vertically stratified fields in the high‐index regions, as shown in Figure 2b. When the identical structure is illuminated under normal incidence as in Figure 2c, the resulting field distribution closely matches the Bloch eigenstate. This indicates that the leaky mode excited in the GMR corresponds to a Bloch‐like guided mode state that resonates through coupling with the external radiation field, establishing the characteristic resonance behavior.

**FIGURE 2:**
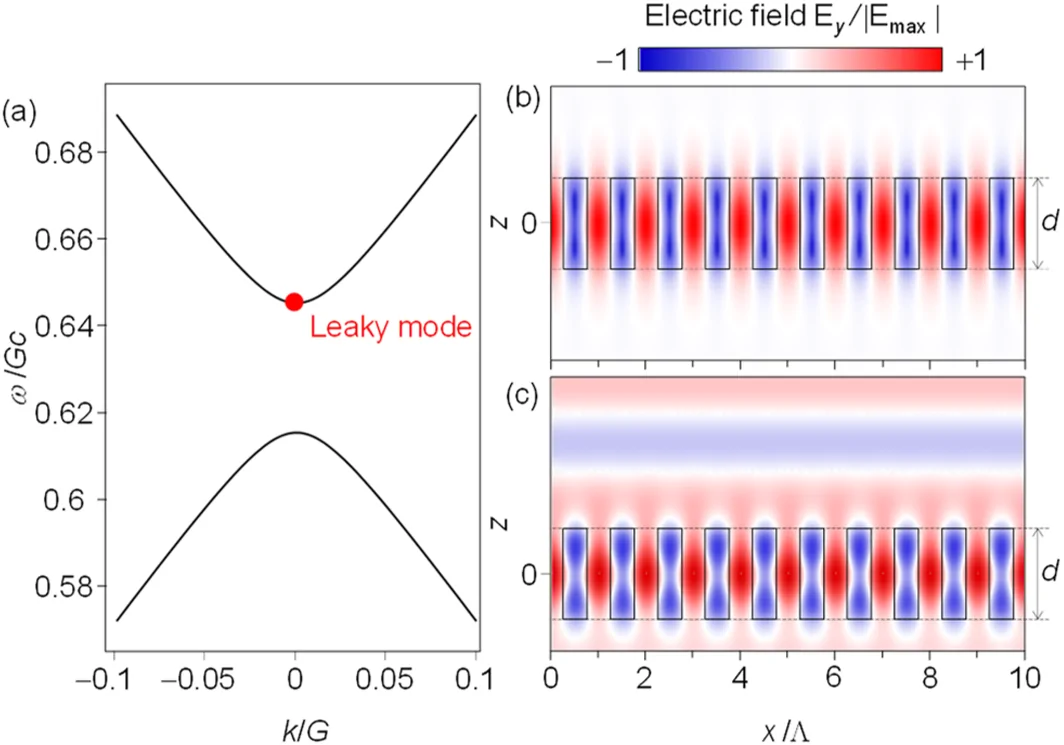
Bloch eigenmode analysis and verification under free‐space excitation. (a) Calculated photonic band structure near the *Γ* point (*k* = 0), highlighting a leaky mode resonance at *λ* = 1.485 μm (red dot) corresponding to the second stop band. Here, *G* = 2π/*Λ* is the grating vector and *c* is the speed of light. (b) Bloch eigenstate field distribution. (c) Field distribution obtained under normal‐incidence plane‐wave illumination as computed numerically.

To further confirm the guided‐mode origin, we analyze the model periodic waveguide with identical parameters using finite‐difference time‐domain (FDTD) simulations. Figure 3a shows the field distribution under single‐edge excitation, where the TE_0_ mode excited in the low‐index segment couples to both TE_0_ and TE_2_ modes in the adjoining high‐index region. Their coherent superposition gives rise to a modulated field profile. The vertical field profile extracted from the grating region is decomposed into slab‐mode components as shown in Figure 3b, where the total field (red line) is accurately reconstructed by the superposition of TE_0_ (black solid) and TE_2_ (black dashed) components. When both edges are simultaneously excited, counter‐propagating guided modes form a standing‐wave pattern equivalent to a Bloch state. Figure 3c shows the resulting field distribution, whereas Figure 3d compares the lateral field cross‐section at *z* = 0 for the dual‐edge excitation (black solid) with the Bloch eigenstate from Figure 2b (red dashed) and the free‐space‐excited GMR field from Figure 2c (blue dashed). Their close agreement verifies that the GMR fields are indeed Bloch‐mode‐like and originate from guided‐mode interference.

**FIGURE 3:**
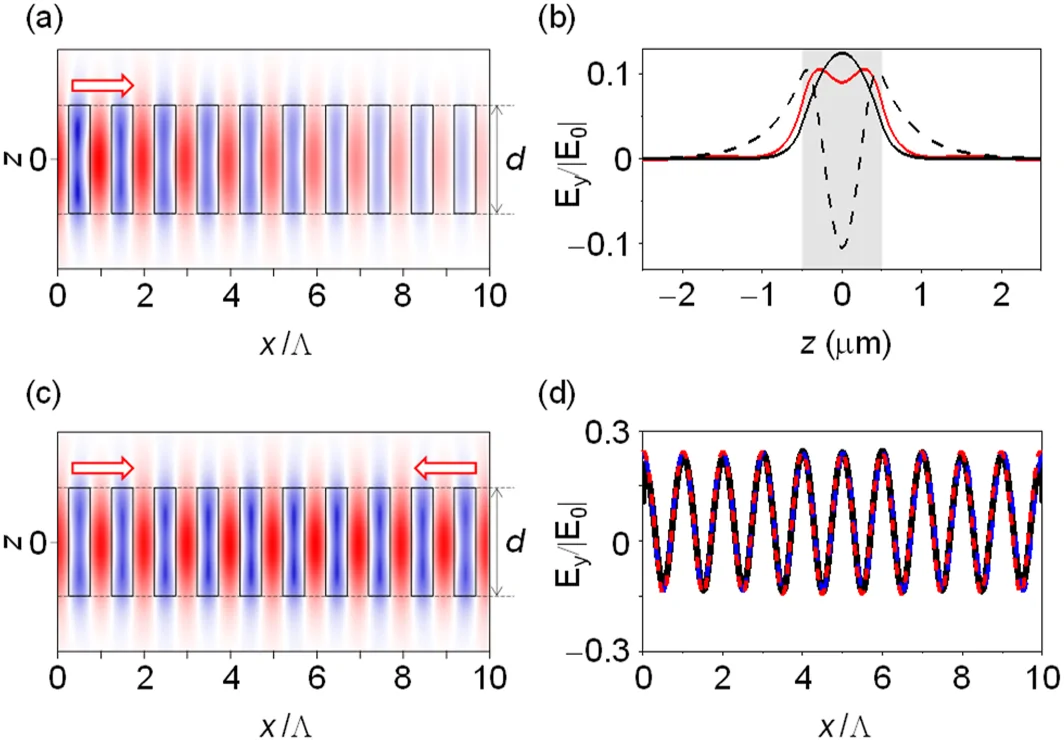
Modal‐decomposition analysis of guided‐mode superposition. (a) Field distribution in the periodic slab waveguide under single‐edge excitation calculated by FDTD, showing field confinement within the high‐index regions. (b) Modal expansion of the field profile where the total field (red) is reconstructed by superposition of TE_0_ (black solid) and TE_2_ (black dashed) components. (c) Dual excitation producing counter‐propagating guided modes that form a standing‐wave pattern equivalent to a Bloch state. (d) Lateral cross‐section field profile at *z* = 0.

## 3 Mie Resonance in Photonic Lattices

Next, we address lattices built with classic particles that are Mie resonant in isolation. Thus, Figure 4 illustrates models and spectra pertaining to Mie resonance in isolated particles and guided‐mode resonance in periodic lattices. The model particle is an infinite circular cylinder with diameter *D* and refractive index *n* placed in air as in Figure 4a. The lattice is an array of identical particles with period *Λ* as per Figure 4b. The lattice operates in the subwavelength regime such that only the zero‐order reflectance (*R*_0_) and zero‐order transmittance (*T*_0_) propagate broadside. The illuminating plane wave is at normal incidence. As is traditional in diffraction and waveguide optics, we define the TE polarization state as having the electric field vector parallel to the particle axis. Figure 4a presents the the total scattering cross‐section (TSCS) of a single particle with *D* = 250 nm and refractive index *n* = 3.5. The magnetic dipole (MD) resonance appears near *λ* = 1.2 μm. Figure 4b shows the reflectance at two selected periods. At *Λ* = 1.1 μm, we have *R*_0_ = 1 and *T*_0_ = 0, that is, perfect reflection, with particle and lattice resonance wavelengths coinciding. Thus, we have local and lateral mode matching, or Mie modal memory, as detailed in ref. [16]. In total contrast, for *Λ* = 0.5 μm, at the particle resonance wavelength, we have *R*_0_ = 0 and *T*_0_ = 1, that is, perfect transmission away from lattice resonance. Obviously, then, the resonance physics and spectra are dominated by the lattice and not the particle.

**FIGURE 4:**
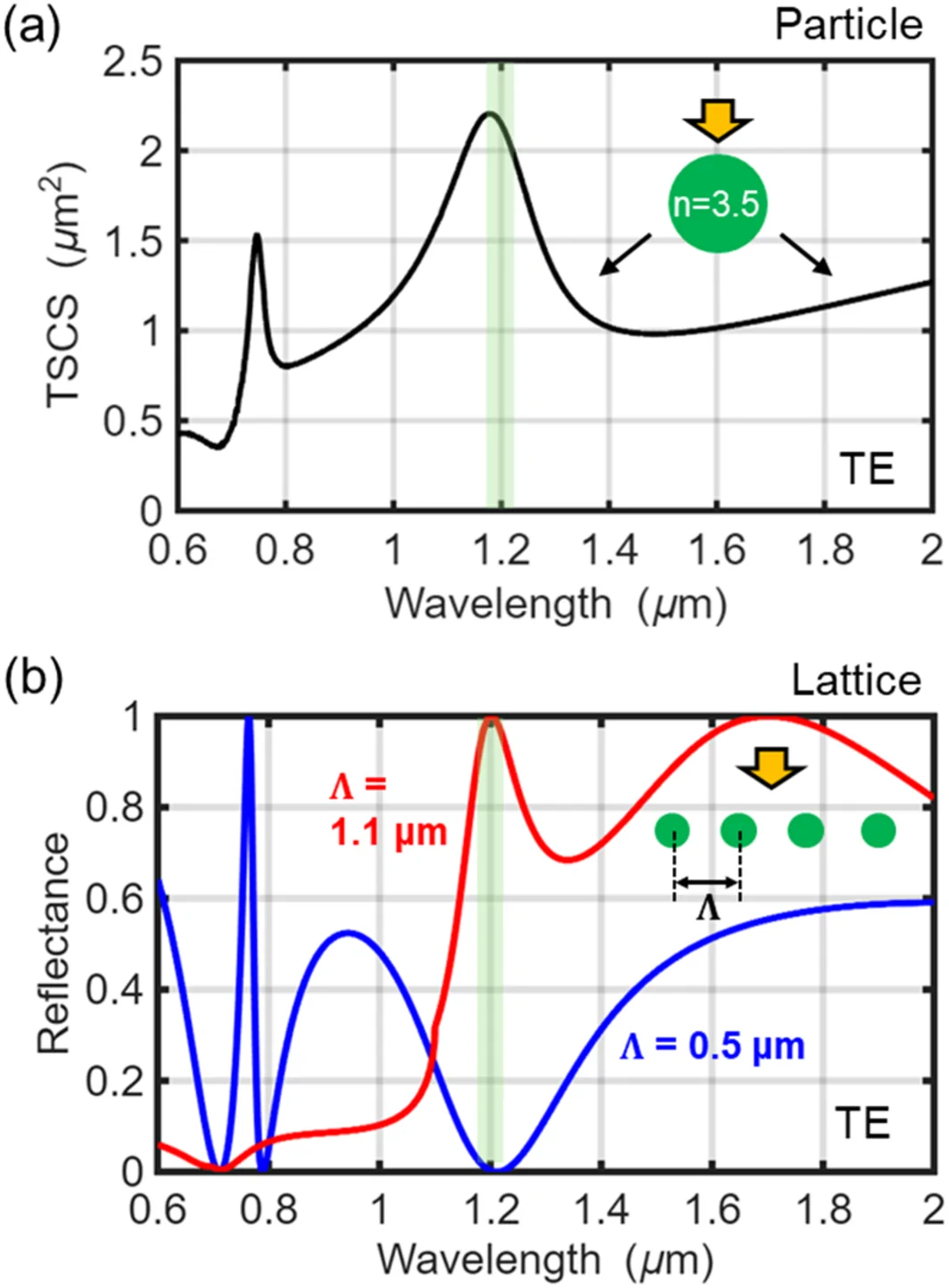
Comparison of single‐particle resonance and lattice‐resonance spectra. (a) FDTD‐computed TSCS spectra under TE polarized light. (b) Zeroth‐order reflectance (*R*_0_) spectra for two lattice periods. The building block particle rod has diameter *D* = 250 nm and refractive index *n* = 3.5 denoting silicon.

Elaborating with FDTD simulations, we treat the same PhL built now with a finite particle set spanning *N* = 1 to *N* = 6. Figure 5 shows the far‐field radiation patterns of the scattered fields at the Mie resonance wavelength *λ* = 1.2 μm with *Λ* = 1.1 μm, which corresponds to perfect reflection *R*_0_ = 1 in the case of the infinite lattice in Figure 4b. It is noted that these distributions represent the total field obtained from the FDTD simulations, including the external illumination, rather than the scattered field of the *N*‐particle system alone. The reflection along the normal direction builds up faithfully as *N* varies from 1 to 6 approaching the limit *R*_0_ = 1. This shows how particle resonance transforms into lateral leaky Bloch modes generating guided‐mode resonance. Because of the high refractive index, relatively few particles are needed to approximate the infinite PhL case. Thus, with sufficient particle count, the PhL appears “periodic”, allowing phase matching of the evanescent first (*m* = ± 1) diffraction orders to the leaky modes supportable by the lattice and leading to GMR and perfect reflection as usual [4]. It is interesting to note that even for *N* = 3, the resonance system has essentially abandoned the resonance characteristics of the single Mie particle.

**FIGURE 5:**
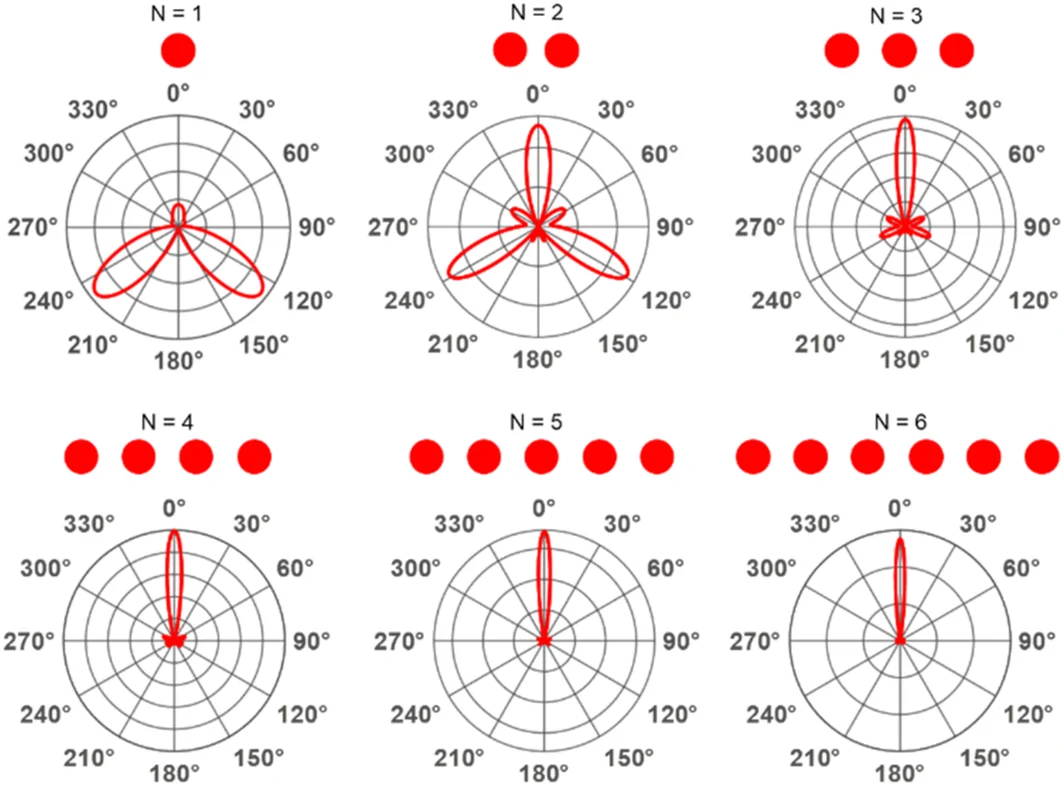
Scattered field distributions at the Mie resonance wavelength *λ* = 1.2 μm with period *Λ* = 1.1 μm. As N increases, perfect reflection *R*_0_ = 1 ensues caused by lattice GMR.

Similarly, for *Λ* = 0.5 μm, at the particle resonance wavelength with *R*_0_ = 0 and *T*_0_ = 1, the perfect transmission in Figure 4b can be modeled. The results are shown in Figure 6, indicating buildup to perfect transmission with an increasing particle count. In particular, the strongest transmission for multiple particle cases (*N* ≥ 2) is at 180° in the forward direction where no power is delivered from a single isolated particle. Importantly, the radiation pattern even in the two‐particle case cannot be built up by a linear superposition of multiple single‐particle fields in the absence of inter‐particle interactions. The perfect transmission at *Λ* = 0.5 μm away from GMR is consistent with effective‐medium theory (EMT). Using the full Rytov eigenvalue EMT formulation [18, 19], the effective index of the film is ∼2.33 yielding zero transmittance near the particle Mie resonance wavelength. The reflectance spectrum of the homogenized lattice is given in Figure 7 showing a null reflectance at *λ* = 1.2 μm. Although the Rytov index varies with wavelength, here, we approximate it as a constant in the neighborhood around the MD resonance.

**FIGURE 6:**
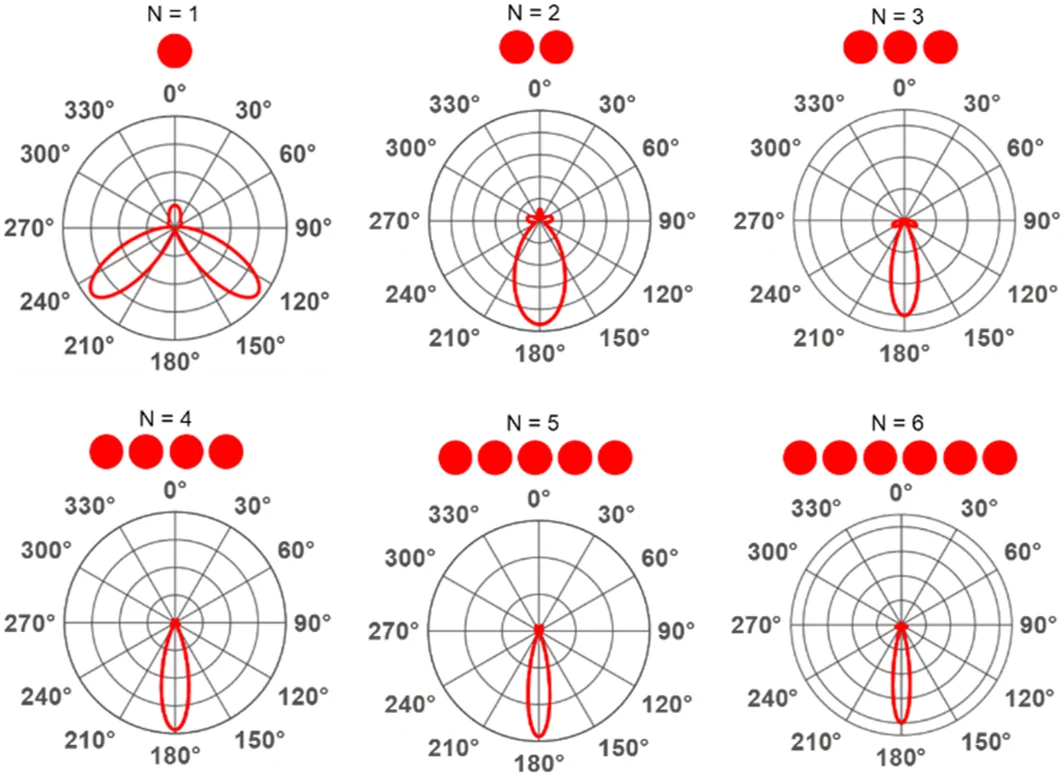
Scattered field distributions at the Mie resonance wavelength *λ* = 1.2 μm but with period *Λ* = 0.5 μm. As *N* increases, perfect transmission *T*_0_ = 1 ensues.

**FIGURE 7:**
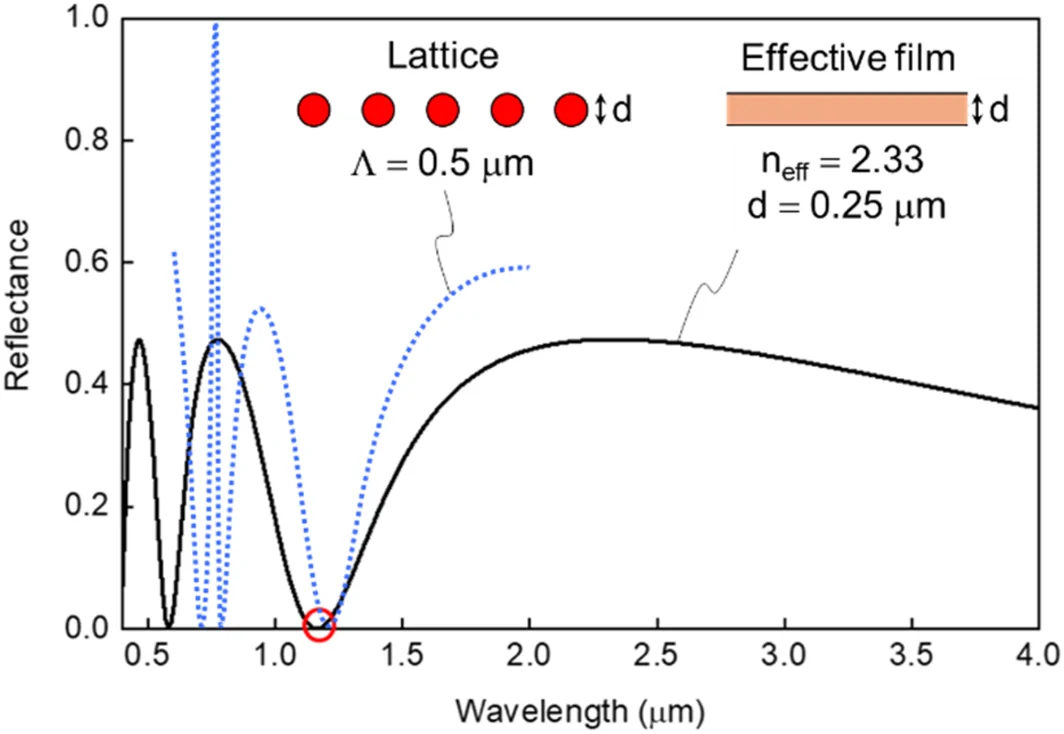
The reflectance spectrum of the homogenized lattice with period *Λ* = 0.5 μm showing antireflection and full transmission near the Mie resonance wavelength. Comparison with the exact numerical result in Figure 4b is included.

## 4 Effect of Mie/FP Resonance Quenching

We now consider if it is possible to quantitatively distinguish the contributions of Mie and vertical FP resonances from GMR spectra. Accordingly, Figure 8 presents a reflectance spectrum of a resonant grating as a function of slab thickness *d* with device parameters as given above in Section 2. In Figure 8a, the yellow dotted line represents the FP dispersion calculated via the Rytov effective‐medium approximation of the PhL [19, 20, 21], whereas the white dashed line denotes the GMR dispersion. The analytical FP maximum matches the numerically computed background spectrum as seen in the figure. The GMR resonance locus appears in the FP background without any consequential relationship seen. Indeed, the GMR locus cuts both high and low FP striations with variation in resonance linewidth due to variation in the lattice thickness as shown in Figure 8a. At a thickness of *d* = 1 μm (red dot), the resonance falls on a high FP locus and exhibits a non‐Lorenzian spectral shape. We note that the resonance linewidth is narrow but not zero as it would be in a perfect bound state in the continuum (BIC) cases [22]. These calculations are done with rigorous coupled‐wave theory (RCWA) [23, 24].

**FIGURE 8:**
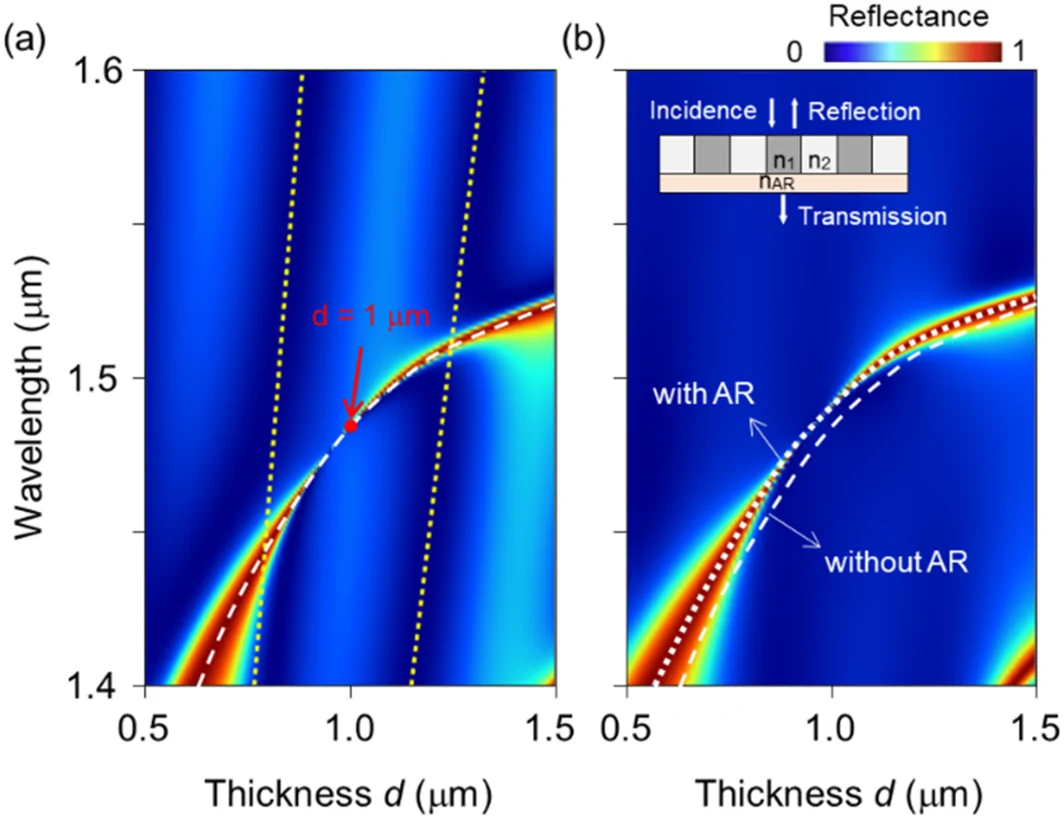
(a) Reflectance spectrum for the resonance lattice with Fabry–Perot dispersion curve noted as yellow dotted lines. White dashed line represents the peak of the guided‐mode resonance locus. (b) Reflectance of the lattice with an added AR layer as illustrated by the inset. The dashed line shows the GMR branch without the AR layer and the dotted line shows the corresponding branch with the AR layer.

We further analyze the structure by introducing a single‐layer anti‐reflection (AR) film beneath the lattice. This modification is intended to suppress and minimize any residual FP effects and accentuate the guided‐mode contribution. As shown in Figure 8b, the FP features are effectively reduced with the GMR signatures dominating. The white dotted line indicates the GMR dispersion in the presence of the AR film, whereas the white dashed line shows the case without it. A slight spectral shift is observed due to the change in the effective modal refractive index introduced by the AR layer. However, the overall resonance trend remains minimally affected. In Figure 8b, the refractive index of the AR coat is *n*_AR_ = 1.320 and the quarter‐wave AR‐film thickness is 281.2 nm with a schematic rendered as an inset. The index is computed as *n*_AR_ = *n*_eff_^1/2^ based on the effective‐medium approximation of the modulated film.

Finally, we provide an example design wherein local particle‐based resonances are essentially annihilated. This is implemented by adding a connective sublayer having identical refractive index as that in the grating ridges as noted in Figure 9. An ideal AR coat is then placed on the output interface of the sublayer. If the ridge is viewed as a particle, this method effectively eliminates the output interface of each particle preventing second‐interface reflection and local Mie type resonance. Hence, FP and Mie resonances in the PhL constituent unit cell medium will be diminished. Nevertheless, the resonance spectrum persists as seen in Figure 9a with characteristic features similar to those in Figure 8. The field structure exhibited in Figure 9b belongs to the laterally propagating left/right Bloch TE_0_ type modes induced by coupling to the ± 1 evanescent diffraction orders. The resulting standing‐wave modal field now locates predominantly in the homogeneous layer with minimal near‐field signatures in the particle array.

**FIGURE 9:**
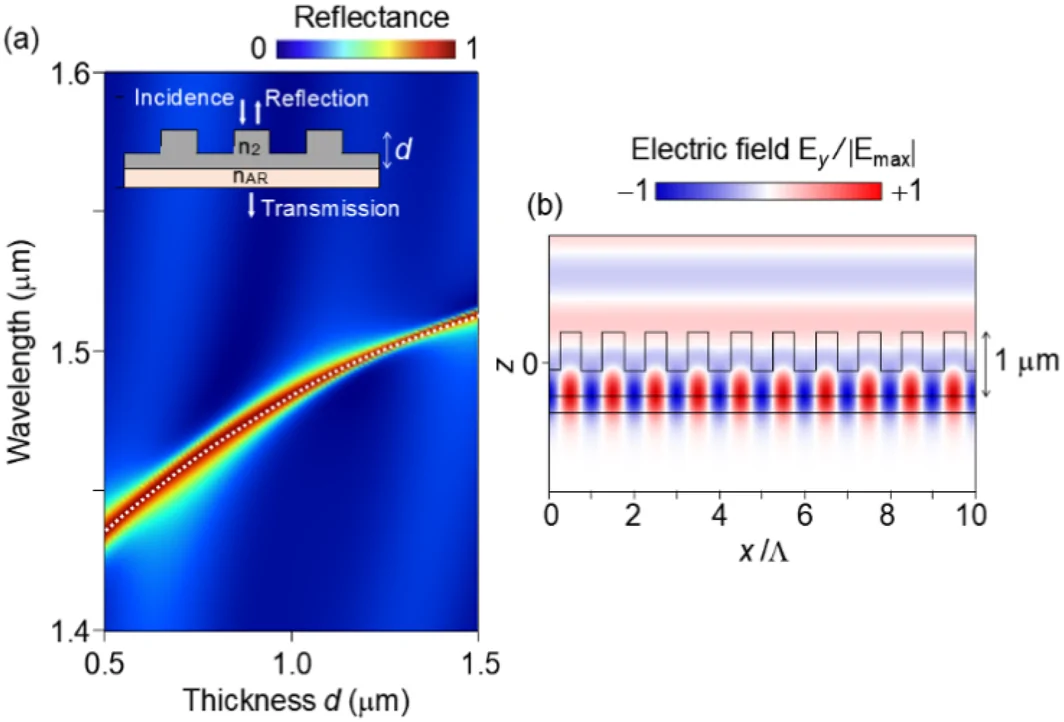
(a) Reflectance spectrum of an example resonant PL incorporating an index‐matched continuum sublayer and simultaneously having an AR layer beneath the sublayer as shown in the inset. The white dotted line represents the guided‐mode resonance peak at the reflectance locus maximum. (b) Bloch mode field distribution at GMR for thickness set as *d* = 1 μm implementing a standing wave pattern due to counterpropagating leaky modes. The near‐field distribution is located away from the grating ridges.

## 5 Discussion

In this work, we have clarified the physical mechanisms governing the resonance properties of one‐dimensional periodic photonic lattices and resolved long‐standing ambiguities regarding the relative roles of Fabry–Perot (FP), Mie, and guided‐mode resonance (GMR) effects. Through rigorous numerical simulations, modal decomposition, and comparative structural designs, we have shown that the electromagnetic field distributions and spectral characteristics commonly attributed to FP or Mie resonances are, in fact, fundamentally explained by the coherent superposition of guided slab modes forming lateral leaky Bloch states.

Among the results of this study is the demonstrated equivalence between edge‐excited guided modes and free‐space‐excited GMR fields. Specifically, counterpropagating guided modes generated under dual‐edge excitation produce standing‐wave field distributions that closely replicate Bloch eigenstates observed under normal‐incidence illumination. This establishes a unified modal picture in which both excitation scenarios access the same underlying leaky‐mode physics. The vertically stratified field patterns observed within high‐index regions—often interpreted as signatures of FP or Mie resonances—are therefore more accurately understood as interference patterns arising from the superposition of even‐parity guided modes (e.g., TE_0_ and TE_2_).

We further examined lattices composed of particles that individually support strong Mie resonances. By varying the lattice period while keeping the particle geometry fixed, we showed that the spectral response is dictated primarily by the lattice period and effective‐medium properties rather than by intrinsic particle resonances. In particular, the same particle resonance wavelength can correspond to either perfect reflection or perfect transmission depending solely on the lattice periodicity. This demonstrates that the dominant resonance mechanism is collective and nonlocal in nature. Although localized particle resonances may influence field distributions through what has been termed “Mie modal memory,” they do not constitute the causal origin of the observed spectra.

The transition from isolated‐particle behavior to collective lattice resonance was further elucidated through finite‐size studies. As the number of particles increases, the system rapidly evolves from independent scattering behavior to a regime characterized by coherent diffraction and coupling to lateral leaky modes. Even a small number of periods can be sufficient to approximate the infinite‐lattice response, highlighting the robustness of the Bloch‐mode formation process. This provides important physical insights into how localized scattering processes transit to diffractive eigenstates capable of supporting high‐Q resonances and perfect reflection.

To isolate and quantify the influence of FP and Mie effects, we introduced structural modifications designed to suppress these mechanisms. Incorporation of antireflection layers effectively minimizes vertical FP resonances while preserving the primary GMR spectral features, demonstrating that FP contributions mainly serve as a weak background modulation. Similarly, by introducing index‐matched sublayers that eliminate internal particle interfaces, we suppress local Mie‐type resonances. In both cases, the principal resonance spectra remain essentially unchanged. These results provide strong evidence that neither FP nor Mie resonances are required for the existence of high‐efficiency lattice resonances and that their roles are secondary to the dominant guided‐mode coupling mechanism.

Taken together, these findings establish a consistent and physically grounded interpretation of resonant photonic lattice behavior: the essential mechanism is the excitation of lateral leaky Bloch modes via phase‐matched diffraction and their subsequent reradiation into free space. FP resonance contributes to the formation and weak modulation of these modes through effective‐medium behavior, whereas Mie resonance may imprint local field characteristics without determining the global spectral response. This unified perspective reconciles disparate interpretations in the literature and aligns with the foundational theory of guided‐mode resonance.

## 6 Conclusions

The implications of this work are significant for the design and optimization of photonic devices based on periodic structures, including metasurfaces, filters, reflectors, and sensors. By emphasizing the primacy of lattice geometry and modal coupling over localized particle resonances, designers can more systematically engineer spectral responses through control of periodicity, symmetry, and effective refractive index. Furthermore, the demonstrated independence of resonance behavior from local particle resonances opens new avenues for simplified and more versatile device architectures, including those incorporating nonresonant or homogenized building blocks.

Future work may extend these concepts to two‐dimensional lattices, explore polarization‐dependent effects, and formulate the connection between leaky‐mode resonances and bound states in the continuum. Additionally, experimental validation of the resonance‐quenching strategies presented here would further strengthen the modal interpretation advanced in this study. Overall, the results presented provide a rigorous and unifying framework for understanding and exploiting resonance phenomena in photonic lattices and metamaterials.

## Author Contributions

**Yu Sung Choi:** investigation, writing – original draft, methodology, validation, visualization, formal analysis. **Keisuke Ozawa:** investigation, writing – original draft, methodology, validation, visualization, formal analysis. **Jae Woong Yoon:** writing – review and editing, investigation, methodology, funding acquisition. **Junichi Inoue:** supervision, project administration, funding acquisition. **Shogo Ura:** supervision, project administration, funding acquisition. **Yeong Hwan Ko:** conceptualization, investigation, methodology, validation, visualization, writing – review and editing, formal analysis. **Robert Magnusson:** funding acquisition, writing – original draft, conceptualization, writing – review and editing, methodology, project administration, supervision.

## Funding

This research was supported by the Leader Researcher Program with Grant No. NRF‐2019R1A3B2068083. Additional support was provided by JSPS KAKENHI (Grant No. JP25KJ1694). Funding was also provided by the Texas Instruments Distinguished University Chair in Nanoelectronics endowment.

## Conflicts of Interest

The authors declare no conflicts of interest.

## Data Availability

Data underlying the results presented in this paper are not publicly available at this time but may be obtained from the authors upon reasonable request.
